# Molecular-Scale Insights into the Interactions between Perfluoroalkyl Substances and Polyethylene

**DOI:** 10.1021/acs.jpcb.5c06774

**Published:** 2026-03-05

**Authors:** Dandara Freitas Thomaz, Eduardo Rocha de Almeida Lima, Nathalia Salles Vernin

**Affiliations:** Chemical Engineering Graduate Program, 28130 Rio de Janeiro State University, Rio de Janeiro, RJ 20550-900, Brazil

## Abstract

Microplastics (MPs) and per- and polyfluoroalkyl substances (PFAS) are two classes of highly persistent contaminants that frequently co-occur in the environment, raising concern about potential synergistic effects. To better understand their interactions, we investigated the adsorption of perfluorooctanoic acid (PFOA) and perfluorooctanesulfonic acid (PFOS) on polyethylene (PE) through molecular dynamics (MD) simulations. The potential of mean force (PMF) at infinite dilution was calculated for both the semicrystalline and crystalline PE models. For semicrystalline PE systems, the PMF minima were −26.5 ± 4.8 kJ mol^–1^ for PFOA and −43.9 ± 4.3 kJ mol^–1^ for PFOS, whereas, for crystalline PE, the values were −26.6 ± 5.2 and −42.0 ± 7.7 kJ mol^–1^, respectively. These results indicate that, within statistical uncertainty, no significant differences are observed between the two PE morphologies for either PFAS when considering the depth of the free-energy minimum. Moreover, PFOS exhibited stronger interactions with PE than PFOA. This behavior reflects not only differences in fluoroalkyl chain length but also the distinct chemical nature of the functional groups, with the larger and more hydrophobic sulfonate headgroup of PFOS compared to the carboxylate group of PFOA. In addition to adsorption strength, molecular orientation at the PE–water interface was characterized. PFAS tails showed a general tendency to align parallel to PE chains within the polymer slab, but this alignment was disrupted upon the transition into water. Notably, PFOS interacting with semicrystalline PE exhibited orientation changes with transitions between parallel and perpendicular alignment associated with local PMF barriers. These orientation-dependent interactions highlight the importance of both chain packing and functional group chemistry in driving PFAS–polymer affinity. Taken together, these findings provide molecular-scale evidence that microplastics can act as reservoirs for PFAS, potentially enhancing their environmental persistence and transport.

## Graphical abstract

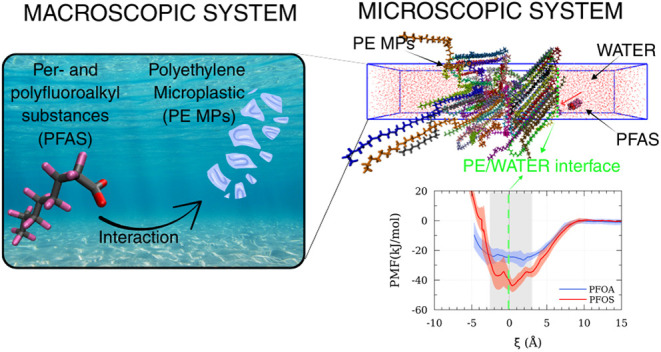

## Introduction

Plastics are widely used for their lightness, low cost, and versatility. Among them, polyethylene (PE) is one of the most common polymers used in commercial applications. However, plastics can take centuries to degrade, making proper disposal a major environmental concern.^1^ Smaller particles, known as microplastics (MPs), have attracted increasing attention from scientists, policymakers, and the public due to their potential health risks.^2^

MPs are particles smaller than 5 mm that can originate from either a primary or a secondary source. Primary MPs are those intentionally produced within this size range and are used to compose, for example, cosmetics and other personal care products. On the other hand, secondary MPs result from the improper disposal of larger plastic materials that undergo wear and tear through mechanical actions, UV radiation, and other environmental factors, ultimately fragmenting them.^3,4^

Per- and polyfluoroalkyl substances (PFAS), commonly referred to as “forever chemicals” due to their exceptional chemical stability, are another class of persistent contaminants, with over 1400 identified compounds used in applications ranging from textiles to firefighting foams.^5^ Despite restrictions on some PFAS under the Stockholm Convention, their environmental levels remain high due to replacement with short-chain analogs, which, while less bioaccumulative, are still highly persistent.^6,7^

In the environment, microplastics and PFAS are ubiquitous,^8^ raising concerns due to their potential interactions.^9,10^ Experimental studies have reported the adsorption of PFAS, including perfluorooctanoic acid (PFOA) and perfluorooctanesulfonic acid (PFOS), onto pristine and aged PE microplastics.^11–17^

These pollutants represent a threat with documented impacts on ecosystems and potential risks to human health. PFAS can interfere with biological processes, such as vitamin D receptor activity,^18^ and have been detected in prenatal exposures worldwide.^19^ MPs, in turn, have been detected in blood, placenta, lungs, and other human tissues, with potential links to cancer and other diseases.^20,21^ Moreover, the combined exposure has been linked to tissue damage, metabolic disorders, neurotoxicity, renal toxicity, liver damage, and reproductive issues.^9,22,23^ Furthermore, it has been shown to alter microbiome structure and increase greenhouse gas emissions in wetlands.^22^ For example, Wu et al.^9^ reported synergistic and antagonistic toxic effects in zebrafish depending on exposure duration, while Zhou et al.^22^ demonstrated that PFAS–MP mixtures disrupted nitrogen cycling by modifying the abundance of ammonia-oxidizing bacteria. These findings underscore the significance of understanding the molecular mechanisms that govern the PFAS–MP interactions.

Molecular simulations, particularly molecular dynamics (MD), offer a powerful approach to investigate these interactions at the molecular level, linking microscopic properties to macroscopic behavior.^24,25^ By sampling system trajectories under controlled conditions, MD enables the prediction of both thermodynamic and dynamic properties.^24^ Previous MD studies have successfully investigated the adsorption mechanisms of contaminants, such as hormones, antibiotics, and pharmaceuticals, on different types of MPs, providing molecular-level explanations for experimental trends.^26–30^ For example, Leng et al.^26^ used MD to determine whether the adsorption of 17β-estradiol occurred on the surface or within the microfibers of MPs such as PE, polypropylene (PP), and polystyrene (PS). Similarly, Chen et al.^27^ investigated the adsorption mechanism of tetracyclines on PE-based MPs, highlighting the crucial role of van der Waals interactions in the process. Extending the scope to other polymers, Liu et al.^28^ explored the interactions between dispersants and microfibers of PS, polyethylene terephthalate (PET), and polyvinyl chloride (PVC). Sahnoune et al.^30^ investigated the sorption of diazepam and paracetamol to PE and PVC. In a more detailed approach, Su et al.^29^ developed a methodology based on MD results to calculate the adsorption equilibrium constant between organic contaminants and PE microplastics.

Concerning PFAS, recent efforts include the work of Wang et al.,^31^ who explored the interactions of fluorotelomer alcohol (FTOH), PFOA, and PFOS with montmorillonite, PE, and PP. However, their study considered only the neutral forms of these compounds in vacuum, neglecting water, and that perfluoroalkyl acids predominantly exist in anionic form at neutral pH due to their very low p*K*_a_. This limits the environmental relevance of their results. Similar methodological approaches have been reported by Enyoh et al.,^32,33^ who combined Grand Canonical Monte Carlo with MD to study PFAS adsorption optimization. While informative, these studies underscore the need for more realistic simulations that incorporate aqueous environments and relevant protonation states.

To address this knowledge gap, this study investigated the molecular mechanisms underlying PFAS–PE interactions by focusing on two representative compounds: PFOA and PFOS. We employed MD to calculate the potential of mean force (PMF) between PE and PFOA/PFOS in very diluted amounts, assess the influence of distinct PE crystalline structures on these interactions, and analyze the orientation of the interaction. Collectively, these objectives provide molecular-level insights into PFAS–PE interactions that can help bridge the gap between atomistic mechanisms and environmental behavior.

## Methodology

### General Remarks on Force Field

The SPC/E force field was employed for water,^34^ while the OPLS-AA force field was used for PE, PFOA, and PFOS.^35,36^ PE parameters were generated with PolyParGen,^37^ and PFOA and PFOS parameters with LigParGen.^38–40^ LigParGen is a web-based parametrization platform developed by the Jorgensen group, which assigns OPLS-AA parameters using the BOSS program. It applies the standard OPLS-AA functional form, incorporating documented updates and extensions to the force field.^40,41^

Both PFOA and PFOS were modeled in their deprotonated (anionic) form. Although reported p*K*_a_ values for these compounds vary across studies, their predominant state at neutral pH is unquestionably the anionic form. Di Battista et al.^42^ classified both as strong acids, with p*K*_a_ values of −0.2 (PFOA) and −3.3 (PFOS). Kutsuna and Hori^43^ reported a range of −0.5 to 2.8 for PFOA and, based on Henry’s constant experiments, suggested a likely p*K*_a_ of 1.3 at 298 K. Moody and Field^44^ noted that replacing hydrogen with fluorine in octanoic acid lowers the p*K*_a_ from 4.89 to 2.80.

Although interfacial effects may modify apparent p*K*_a_ values, studies on carboxylic and sulfonic acids indicate that p*K*_a_ shifts at water–organic interfaces are typically limited to approximately one p*K*_a_ unit.^45^ Under nanoconfinement, even smaller increases (∼0.2 to 0.4 p*K*_a_ units) have been reported for simple carboxylic acids such as formic and acetic acid.^46^ These findings support the use of deprotonated PFOA and PFOS as a reasonable approximation under neutral pH conditions.

It is important to note that the partial charges generated by LigParGen led to a total molecular charge that differed marginally from −1 due to numerical rounding. A minimal uniform correction was therefore applied to enforce an exact net charge of −1 for each PFAS molecule. Further details on the force field parameters for PFOA and PFOS are provided in Section 1 of the Supporting Information.

Importantly, no explicit counterion was inserted into the simulation box. The system carries a net charge of −1, which, under periodic boundary conditions with Ewald or particle–particle particle–mesh (PPPM) electrostatics, is compensated by a uniform background plasma.^47^ Given the size of the simulation box (ranging from 26,324 to 26,328 atoms), this corresponds to a very small effective charge density. The simulations were performed in the infinite dilution limit, under which local structural and thermodynamic properties of the solute are not expected to be significantly influenced by specific counterion–solute correlations. Within this framework, the neutralizing background provides a consistent treatment of the electrostatics.

The Lennard–Jones (LJ) potential with a cutoff of 12 Å was used to describe the short-range interactions between atoms separated by three or more bonds. For intramolecular 1–4 interactions, a scaling factor of 0.5 was applied. The geometric combination rule was employed for σ and ϵ.^35^

Electrostatic interactions at distances shorter than 12 Å were calculated directly, whereas contributions beyond this cutoff were computed in reciprocal space using the PPPM method^48^ with a precision of 10^–4^. The same scaling factor of 0.5 applied to 1–4 atom pairs in LJ interactions was also used for the Coulombic terms.

### System Modeling

PFAS molecules are orders of magnitude smaller than PE microplastics, which can reach sizes of up to 5 mm. From the perspective of an individual PFAS molecule, the PE surface can therefore be regarded as effectively infinite and planar.

The simulation boxes were built using Playmol^49^ and Packmol^50^ to mitigate sampling issues and prevent molecule overlap. The Large-scale Atomic/Molecular Massively Parallel Simulator (LAMMPS)^51,52^ software was used to carry out MD. For all simulations, periodic boundary conditions were applied along the *x*, *y*, and *z* axes, and the time step was set to 1 fs.

Two simulation boxes were constructed, each containing 50 PE chains with 36 monomers, differing only in their initial conformations: (i) coiled chains and (ii) extended chains.

For the coiled conformation, the chains in random configurations were inserted into a cubic box of 90 × 90 × 90 Å^3^. The system was simulated in the NpT ensemble at 500 K and 1 atm for 0.5 ns, followed by cooling to 300 K over an additional 0.5 ns. After equilibration, the box dimensions stabilized at approximately 46 × 46 × 46 Å^3^. For the extended conformation, the PE chains were first constructed in a fully stretched configuration and aligned parallel to each other along the *z*-axis before being placed in the simulation box. This initial configuration was then pre-equilibrated for 0.5 ns in the NVE ensemble to relax bond and angle vibrations while preserving the overall extended arrangement. Both systems were solvated by adding 5133 water molecules to each simulation box.

The two systems with different chains were equilibrated until no further significant changes in their conformations were observed, as shown in Figure 1, and the potential energy remained constant. The system with coiled PE chains was equilibrated for 234 ns, yielding a semicrystalline structure, whereas the system with extended chains was equilibrated for 100 ns, resulting in a more crystalline structure. For visualization purposes, PE chains are shown in distinct colors in all figures.

**Figure 1:**
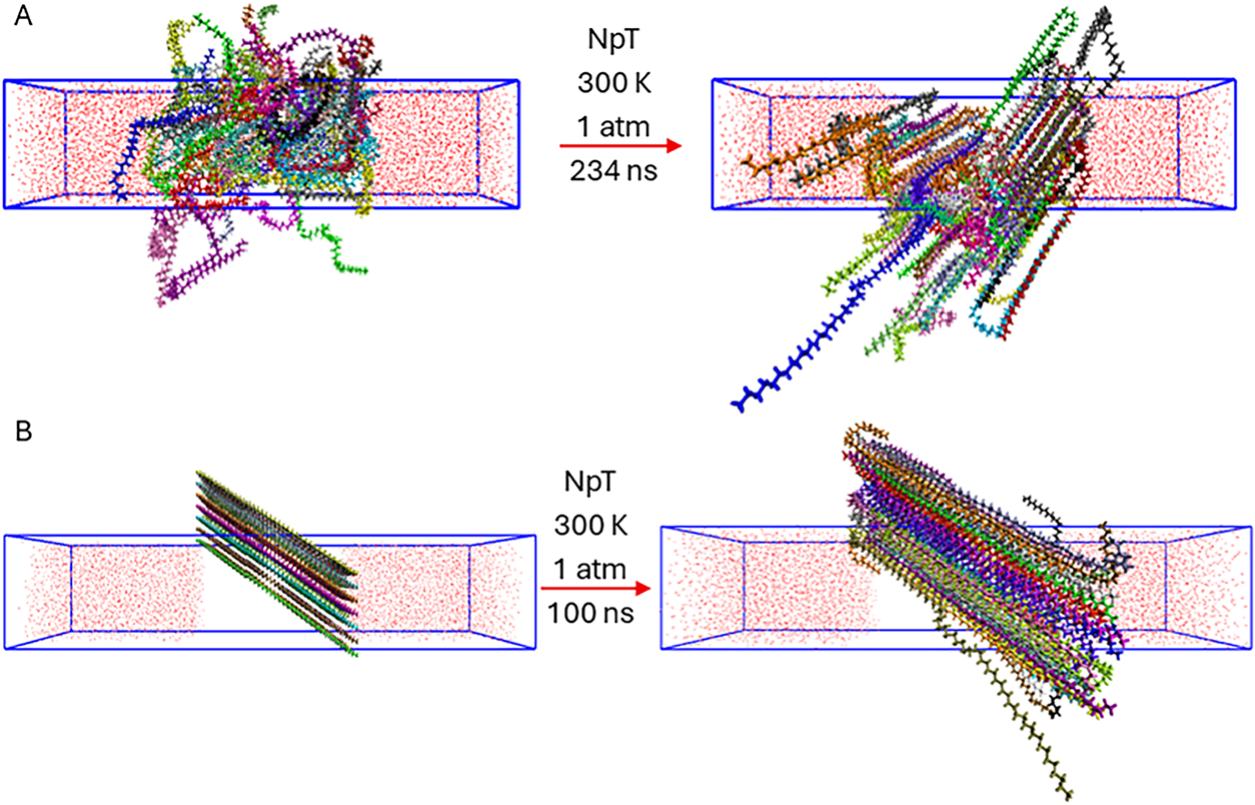
Insertion of water into the (A) semicrystalline polyethylene simulation box and (B) crystalline polyethylene simulation box, followed by equilibration of the systems. The molecular coordinates were wrapped within the central simulation cell while preserving intramolecular connectivity, thereby preventing atoms belonging to the same molecule from being artificially separated across periodic boundaries. Consequently, the apparent “voids” observed within the PE slab are not genuine empty regions. Rather, they correspond to PE atoms whose molecules extend across the boundaries of the central box and are thus represented as adjacent periodic replicas.

After equilibration of the PE models in aqueous medium, PFAS molecules (PFOA or PFOS) were placed approximately 15 Å from the polyethylene–water interface (Figure S3). Simulations were performed in boxes containing either semicrystalline or crystalline PE for equilibration and volume calculation. For semicrystalline PE, equilibration consisted of 1 ns in the *NVT* ensemble followed by 5 ns in the *NpT* ensemble, resulting in box dimensions of approximately 32 × 32 × 180 Å^3^. Crystalline PE was simulated for 5 ns in the *NpT* ensemble, reaching box dimensions of approximately 34 × 34 × 190 Å^3^.

### Computational Analysis

Computational analysis was employed to investigate the molecular-level interaction mechanisms between PFAS and polyethylene. Different tools were applied to extract quantitative information from the simulations, allowing for the interpretation of thermodynamic and structural properties.

#### Umbrella Sampling (US) and PMF

With the contaminants positioned, the objective shifted to bringing them closer to the surface of the PE to obtain free-energy profiles. The potential of mean force (PMF) describes the free-energy variation along a chosen reaction coordinate.^53,54^ Direct calculation is often hampered by insufficient sampling, especially in the presence of high energy barriers.^55^ To overcome this, we applied the umbrella sampling method, where harmonic biasing potentials were introduced along the *z*-distance between the centers of mass of PE and PFAS.

Initially, the PFAS was continuously pulled toward the center of mass of PE, generating initial configurations for each sampling window. The reaction coordinate along the *z*-axis was partitioned into discrete intervals, with each interval defining a sampling window. Within each window, the PFAS contaminants were restrained around the center ξ_*i*_′ of the *i*-th window. The harmonic potential applied in each window is given by eq 1

1 $$w_{i}\left(z\right)=\frac{k}{2}{\left(\xi^{\prime }-{\xi_{i}}^{\prime }\right)}^{2}$$

where *k* is the force constant, ξ′ is the reaction coordinate defined as the *z*-distance between the centers of mass of PE and PFAS, and ξ_*i*_′ is the reference position of window *i*. In the applied US method, a force constant of 10 kcal mol^–1^ Å^–2^ was used, unless otherwise stated. The restraint potentials as a function of ξ′ guide the system from one thermodynamic state to another, resulting in a histogram distribution in each window. Each window was equilibrated for 1 ns, followed by a standard production time of 2 ns. At least four independent simulations were performed for each window in order to quantify the statistical uncertainty and compute the standard deviation of the PMF profile. All pulling and sampling simulations were conducted at 300 K, 1 atm, NVT ensemble, using the Colvars module available in LAMMPS, which enables the application of restraint potentials along the defined path. The NVT ensemble was employed to preserve the interfacial area during umbrella sampling and prevent box-size fluctuations.

The biased histograms from all windows were subsequently combined using the Weighted Histogram Analysis Method (WHAM)^56,57^ to obtain the unbiased PMF profile. This approach allows for reconstruction of the free-energy landscape with improved statistical convergence.

#### Density Profiles

As mentioned earlier, the original reaction coordinate was defined as the *z*-distance between the centers of mass of PE and PFAS. For postprocessing and more straightforward interpretation, this distance was redefined as ξ, representing the distance of the PFAS molecule from the nearest Gibbs dividing surface (i.e., the PE–water interface) along the *z*-axis.

To determine the Gibbs dividing surface, the atomic density profiles of PE (ρ_PE_) and water (ρ_w_) along *z* were computed and fitted with hyperbolic tangent functions. For the PE phase, the profile was fitted as

2 $$\rho_{\mathrm{PE}}\left(z\right)=\frac{1}{2}\rho_{b,\mathrm{PE}}\tanh\left[\frac{2\left(z-h_{1}\right)}{D_{1}}\right]-\frac{1}{2}\rho_{b,\mathrm{PE}}\tanh\left[\frac{2\left(z-h_{2}\right)}{D_{1}}\right]$$

and for water as

3 $$\rho_{w}\left(z\right)=\rho_{b,w}-\frac{1}{2}\rho_{b,w}\tanh\left[\frac{2\left(z-h_{3}\right)}{D_{2}}\right]+\frac{1}{2}\rho_{b,w}\tanh\left[\frac{2\left(z-h_{4}\right)}{D_{2}}\right]$$

where ρ_b,*i*_ is the bulk density of component *i*, *D* is related to the interfacial width, and *h*_*j*_ to its positions. The Gibbs dividing surfaces were defined as the position where eq 2 equals eq 3.

#### Order Parameter and Angle of Interaction between PE and PFAS

The orientation of the PFAS tail was assessed using the order parameter *S*_*v*_ calculated relative to the resultant vector of the polyethylene chains in the vicinity of the PFAS molecule, as

4 $$S_{v}=\frac{3\left\langle \cos^{2}\theta \right\rangle -1}{2}$$

where θ is the angle between the vector **v**_1_ associated with the PFAS molecule and the vector **v**_2_ associated with the PE chains.

The resultant vector for the PFAS molecule was defined as the sum of the normalized vectors formed between carbon atoms *k* + 1 and *k* – 1 (i.e., carbon 1,3-intramolecular interactions),^58^ excluding the carbon atom of the functional group (Figure 2A), divided by the total number of such vectors

5 $$v_{1}=\frac{1}{n_{c}-2}\sum_{k=2}^{n_{c}-1}\frac{x_{k+1}-x_{k-1}}{\left|x_{k+1}-x_{k-1}\right|}$$

where *n*_c_ is the number of carbon atoms in the tail, excluding the carbon of the functional group, and **x**_*k*_ is the coordinate of the *k*-th atom.

**Figure 2:**
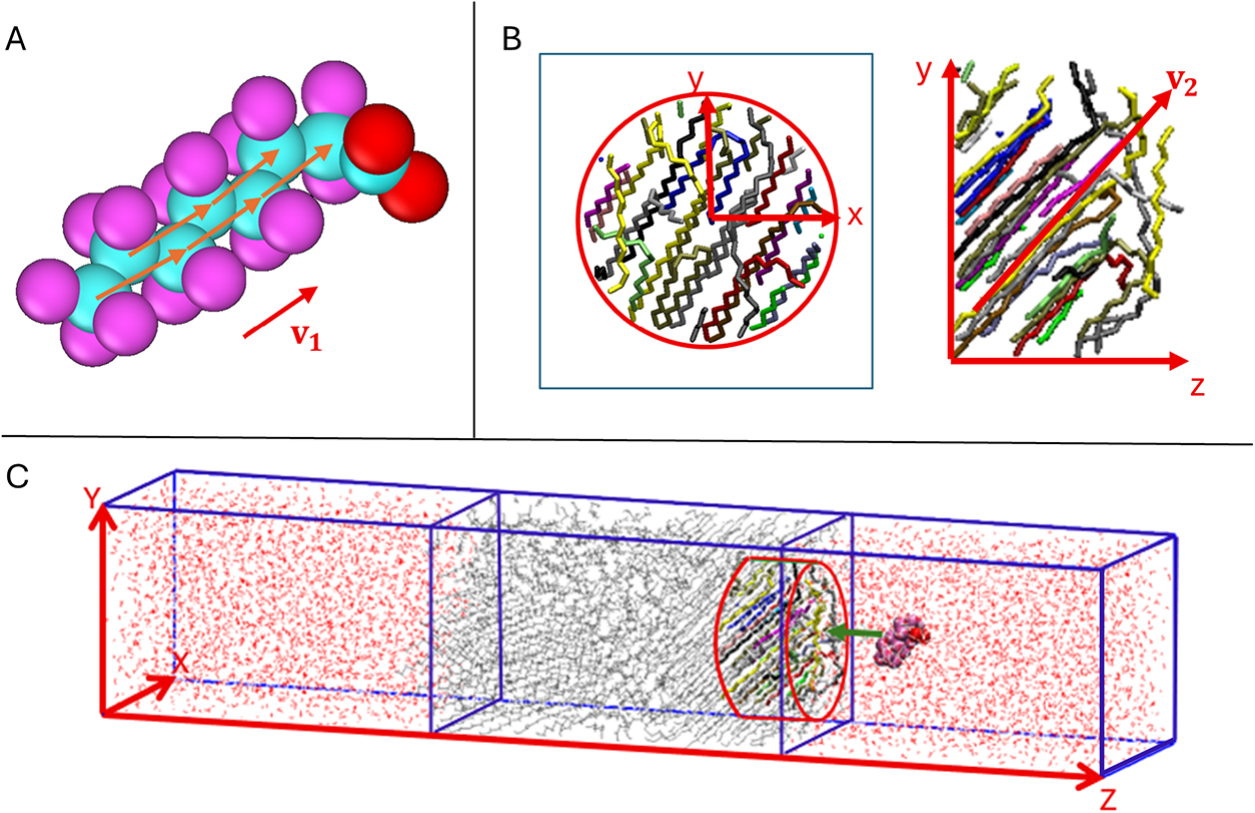
Schematic representation of vector calculations, highlighting (A) a representative vector formed between carbon atoms of PFAS molecules (PFOA or PFOS), (B) a representative resultant vector formed between carbon atoms of PE chains, and (C) the cylindrical sampling region defined within the PE slab. For an improved visualization of the cylinder, molecular coordinates were fully wrapped within the central simulation box.

For the calculation of the vector associated with PE, a cylindrical sampling region was defined as the reference volume around the PFAS molecule (Figure 2C). The cylinder axis was defined as the line passing through the projection of the PFAS center of mass onto the *xy*-plane. The cylinder had a radius of 13 Å in the *xy*-plane (Figure 2B), with its lower boundary in the *z*-direction set 18 Å above the PE center of mass and its upper boundary extending to the PE–water interface (Figure 2C). As in the case of PFAS, for each PE chain fragment within the cylinder, a normalized vector was formed between carbon atoms *k* + 1 and *k* – 1.

To prevent cancellations caused by opposite orientations due to chain folding, each C–C vector was oriented to maximize the magnitude of the resultant vector (reversing its direction when necessary). Finally, **v**_2_ was defined as the sum of these vectors, constrained to form an angle smaller than 90° with the *z*-axis. This constraint was introduced as a convention to ensure consistent interpretation of the ensemble-averaged angles since PE chains are chemically identical at both ends.

#### Root-Mean-Square Deviation (RMSD)

To assess possible intramolecular conformational changes of the PFAS molecules, the root-mean-square deviation was calculated after the removal of overall translation and rotation by aligning each frame of the trajectory to a reference extended conformation. The RMSD is defined as

6 $$\mathrm{RMSD}=\sqrt{\frac{\sum_{i=1}^{N_{\mathrm{atoms}}}\left(x_{i}\left(t\right)-x_{i}^{*}\right)}{N_{\mathrm{atoms}}}}$$

where *N*_atoms_ is the number of atoms included in the analysis, **x**_*i*_(*t*) is the position of atom *i* at time *t* and **x**_i_^*^ denotes its position in the reference structure. The RMSD analysis was performed considering only the carbon atoms of the PFAS backbone, including the carbon atom of the carboxylate group in PFOA and also the sulfur atom of the sulfonate group in PFOS. By restricting the analysis to these atoms, the RMSD specifically captures backbone conformational fluctuations while minimizing contributions from the high-frequency motions of peripheral atoms. RMSD analyses were carried out using the Visual Molecular Dynamics (VMD) software package.^59^

## Results and Discussion

To gain a deeper understanding of the molecular structuring at the interface, we analyzed the density profiles of water in contact with the two models of PE.

In the semicrystalline PE box, the observed densities were approximately 1.04 g·cm^–3^ for water and 0.93 g·cm^–3^ for PE (Figure S4A,B). In the crystalline PE box, the values found were 1.00 g·cm^–3^ for water and 0.99 g·cm^–3^ for PE (Figure S4C,D). This behavior is consistent with the expected trend for PE, where higher crystallinity leads to higher density due to the increased alignment and packing of the polymer chains.^60^

These differences can be further understood by the molecular organization of PE, which is dependent on its production method. According to Yeh et al.,^61^ PE exhibits different chain topologies in the amorphous regions, including tails, bridges, loop segments, and cyclic chains, which directly influence the density values. Moreover, Fu et al.^10^ highlighted that PE typically occurs in a semicrystalline state. In our model, this morphology is represented by lamellar crystalline regions interspersed with amorphous domains, a characteristic feature of high-density polyethylene (HDPE).^57^ Consequently, fluctuations in PE density are expected: smaller variations correspond to a more crystalline arrangement and, therefore, a higher density.

It should be noted, however, that this study did not pursue a detailed analysis of crystallization. The only assertion that can be made is that both models correspond to HDPE, since the initial polymer chains used were nonbranched.

From the data in Figure S4, the Gibbs dividing surface for each system was determined (dashed lines in the figure). This reference was then used to adjust the reaction coordinate of the PMF, the order parameter, angle, and RMSD ensuring a more consistent analysis by shifting the reference point from the PE slab center of mass to the interface.

With this reference established, the interaction between PFAS molecules and the PE surface was investigated through PMFs calculated along the reaction coordinate. Sufficient overlap between the distributions of adjacent umbrella sampling windows was ensured for all four systems. Representative histograms from one set of simulations for each system are provided in the Supporting Information (Figure S5), while additional independent replicas were performed to compute statistical uncertainties. The number of windows in each system was chosen independently to achieve this overlap for each case and to adequately represent the approach of PFAS molecules to the interface (more information can be found in the Supporting Information, Section 4).

Based on the obtained histograms, PMFs were generated using the WHAM method (Figure 3), allowing the observation of the Helmholtz free-energy profile. To enable a direct comparison between the profiles, the zero of the PMF was consistently defined from the plateau region of the curve, corresponding to large PE–PFAS separations where the interaction becomes negligible. Therefore, a constant function was fitted to the last PMF windows, and the resulting average plateau value was subtracted from the entire curve. The values of the free-energy minima are presented in Table 1.

**Figure 3:**
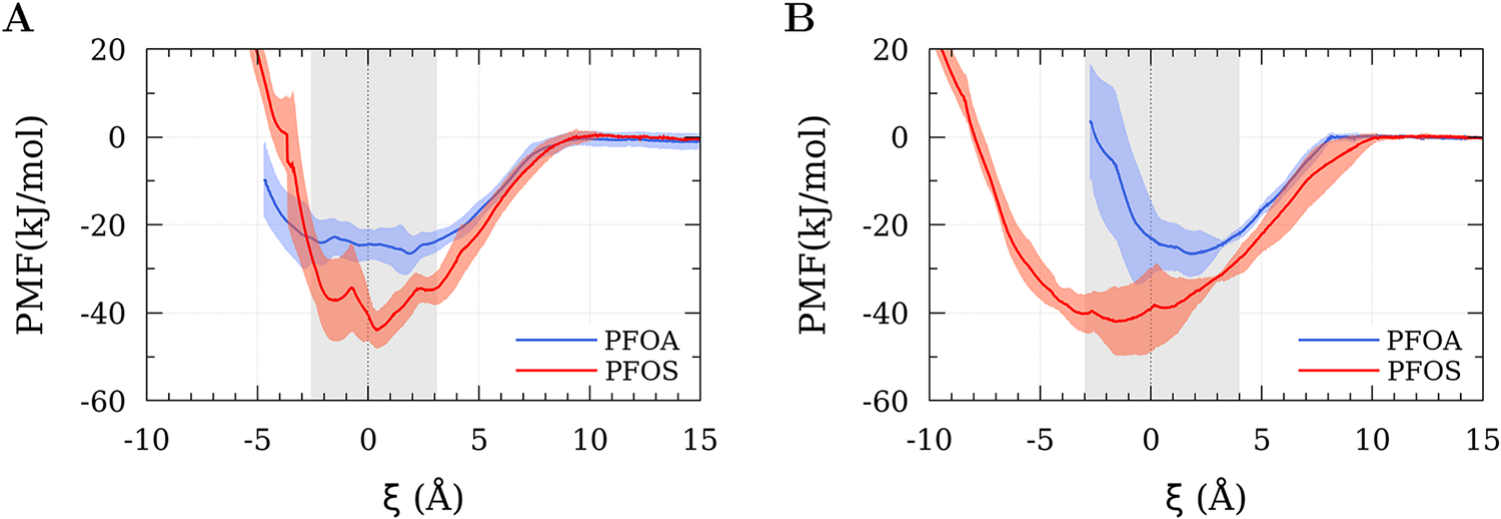
Potential of mean force profiles obtained for PFOA and PFOS interacting with (A) semicrystalline PE and (B) crystalline PE. The dashed line indicates the Gibbs dividing surface, while the gray shaded area represents the interfacial region where PE and water molecules coexist, and the red and blue shaded areas represent the uncertainties of the respective simulation result.

**Table 1:** Free Energy Minima for the Interactions of PFOA and PFOS with Crystalline and Semicrystalline PE

polyethylene	PFAS	minimum value (kJ mol^–1^)
semicrystalline PE	PFOA	–26.5 ± 4.8
semicrystalline PE	PFOS	–43.9 ± 4.3
crystalline PE	PFOA	–26.6 ± 5.2
crystalline PE	PFOS	–42.0 ± 7.7

For both crystalline and semicrystalline PE, anionic PFAS exhibit pronounced attractive free-energy minima at the PE–water interface. For PFOA, the free-energy minimum is −26.5 ± 4.8 kJ mol^–1^ for the semicrystalline PE system and −26.6 ± 5.2 kJ mol^–1^ for the crystalline PE system, while for PFOS the corresponding values are −43.9 ± 4.3 and −42.0 ± 7.7 kJ mol^–1^, respectively (Figure 3). Within statistical uncertainty, no significant differences are observed between the two PE morphologies for either PFAS when considering the depth of the free-energy minimum. Notably, the absence of a measurable dependence on PE morphology is somewhat counterintuitive, as higher crystallinity is commonly associated with reduced free volume and, therefore, weaker adsorption capacity.

Small fluctuations in the PMF profiles are expected even for idealized, uniform interfaces; in the present systems, such variations may be further influenced by the intrinsic roughness of the PE surface at the atomistic scale and by local variations in chain packing and orientation at the interface, which generate heterogeneous interaction environments for PFAS adsorption. Overall, PFAS molecules preferentially accumulate at the PE surface rather than diffuse into the polymer matrix.

For the semicrystalline PE systems, a shallow energy barrier was observed near the Gibbs dividing surface, likely arising from the rearrangement of interfacial water and PE molecules. A similar behavior was reported by Zheng et al.^62^ for PFOA and PFOS interacting with 1-palmitoyl-2-oleoyl-glycero-3-phosphocholine (POPC) lipid bilayers, where the effect was attributed to the presence of a hydration layer. Moving further into the PE slab beyond this barrier, an additional but shallower minimum was identified before the PMF rose again (Figure 3A).

For the crystalline PE, PFOS exhibited a broad energy basin along with a small barrier near the Gibbs dividing surface. However, PFOA displayed a distinct behavior: the unfavorable region for PFOA–PE interactions, unlike that in the other systems, appeared in the interfacial region (shaded area in Figure 3B), limiting its adsorption capacity.

The high hydrophobicity of PE, relative to other common microplastics such as PS and PVC, is expected to further promote PFAS adsorption at the polymer–water interface. This follows the general hydrophobicity trend reported in the literature: PP > PE > PS > PC (polycarbonate) > PVC.^14,23^

When the minimum energy values between PFOA and PFOS for both PE models were compared, PFOS consistently exhibited a lower minimum energy value. Importantly, the stronger free-energy decrease observed for PFOS relative to PFOA (Figure 3) aligns with experimental observations showing greater accumulation of PFOS than PFOA on PE.^11–17^ This behavior may be attributed to a combination of factors, including the longer carbon chain of PFOS (excluding the carbon due to the functional group of PFOA), which enhances hydrophobic interactions, solvation effects, and differences in the terminal functional group. Importantly, our PMF calculations were performed in explicit water. As a result, the reported free-energy profiles capture the competitive balance between PFAS–water and PFAS–PE interactions at the interface, which also reflect the reduced aqueous solubility of PFOS (680 mg L^–1^) relative to PFOA (3400 mg L^–1^).^63^

A similar trend was observed experimentally for chain length effects on PE, where longer perfluorocarboxylic acids (e.g., PFOA) showed greater adsorption than shorter ones (e.g., PFHxA, perfluorohexanoic acid).^64^ Consistent with these observations, studies on PFAS adsorption in kaolinite and tropical soils have also reported stronger adsorption of PFOS compared to PFOA.^65,66^

Moreover, the larger size and the higher electronegativity of the sulfonate group compared to carbonate enhance the hydrophobic character of PFOS during sorption onto MP surfaces. For molecules with the same carbon chain length, this structural distinction explains the stronger sorption typically observed for perfluorinated sulfonates compared to carboxylates.^23,66,67^

Wang et al.^31^ investigated the interactions of fluorotelomer alcohol (FTOH), PFOA, and PFOS with montmorillonite, PE, and PP using MD. Their results showed that FTOH exhibited the strongest adsorption on PE and PP, while PFOS and PFOA preferentially adsorbed onto montmorillonite, with PFOS showing particularly strong interactions attributed to its sulfonate group. Similarly, Enyoh et al.^33^ reported efficient sorption of various PFASs on PE, with the sorption order PFHxA < PFBS (perfluorobutanesulfonic acid) < PFOA < PFNA (perfluorononanoic acid) < PFDA (perfluorodecanoic acid) < PFHxS (perfluorohexanesulfonic acid) < PFOS. Both studies, however, modeled PFAS molecules in their neutral forms and neglected the presence of water, placing the contaminants in a vacuum to interact directly with the surfaces. While this methodological limitation reduces environmental realism, since PFASs exist mainly in their anionic forms at neutral pH and interact in aqueous environments, their findings consistently highlight the strong affinity of PE for long-chain PFASs. Overall, these studies reinforce two key trends: sulfonate headgroups tend to interact more strongly with PE than carboxylates, and chain length remains a dominant factor controlling PFAS sorption.

Considering other contaminants, Sahnoune et al.^30^ reported minimum free energies of adsorption for diazepam interacting with PE(100), PE(010), and amorphous PE of −33.7, −32.7, and −33.4 kJ mol^–1^, respectively. For paracetamol, the corresponding values were −20.0, −25.2, and −20.9 kJ mol^–1^. Additionally, Oliveira et al.^68^ reported adsorption energies of approximately −8 and −23 kJ mol^–1^ for bisphenol A and benzophenone, respectively, interacting with PE nanoplastics. In comparison, PFOS exhibits much stronger interactions with PE, highlighting its exceptionally high affinity for these other organic contaminants.

To gain further insights into the interaction between PFAS and PE, we analyzed the order parameter *S*_*v*_, which quantifies the orientation of the PFAS carbon tails relative to that of the PE carbon chains (Figure 4). For this calculation, the angle was defined between the PFAS vector **v**_1_, pointing from the terminal carbon of the fluoroalkyl tail toward the headgroup (−SO_3_^–^ for PFOS and −COO^–^ for PFOA), and the PE vector **v**_2_, oriented toward the PE–water interface. In this framework, *S*_*v*_ = 1 corresponds to perfect alignment of the two vectors, *S*_*v*_ = −0.5 indicates a perpendicular orientation, and *S*_*v*_ values around zero represent random alignment.

**Figure 4:**
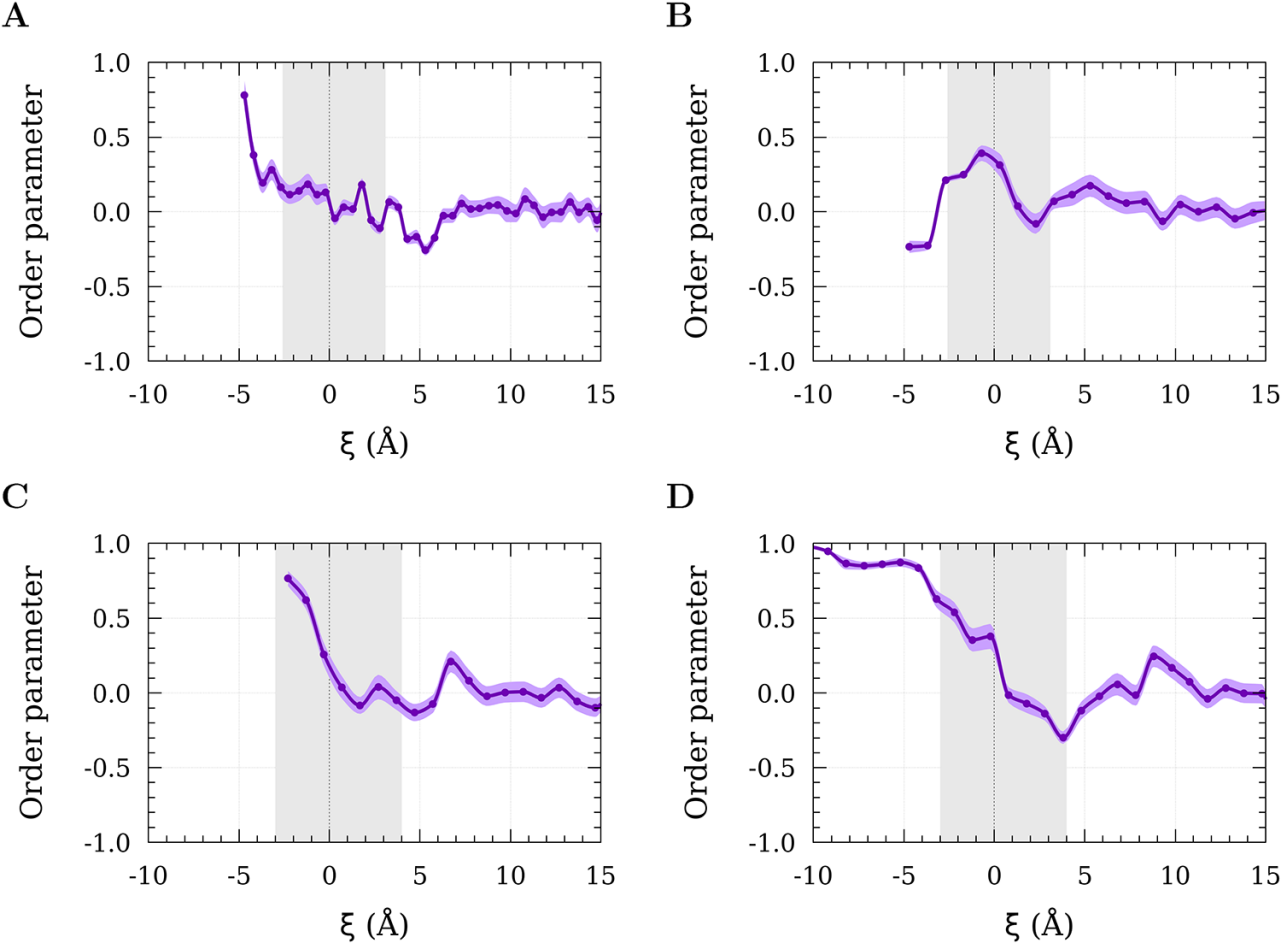
Order parameter for the interaction between (A) PFOA and semicrystalline PE, (B) PFOS and semicrystalline PE, (C) PFOA and crystalline PE, and (D) PFOS and crystalline PE as a function of the reaction coordinate. The dashed line marks the Gibbs dividing surface, while the gray shaded area denotes the interfacial region where PE and water molecules coexist. The purple shaded area represents the 95% confidence interval (*p* = 0.05).

The contaminants exhibited random orientations for all systems far from the PE–water interface (Figure 4), indicating that the PFAS molecules can rotate freely. At these distances, the 95% confidence intervals (*p* = 0.05) were broad (shaded bands), reflecting the high variability in the average orientation. In other words, the biased PMF simulations suggest that all molecular orientations accessible at the largest separation distances remain possible when the molecule interacts with the PE. The confidence intervals narrowed as the perfluoroalkyl substances approached the interfacial region, indicating reduced angular dispersion and a more consistent mean orientation at each position along the reaction coordinate, ξ. Complementary boxplots of the interaction angles as a function of the reaction coordinate are provided in the Supporting Information (Figure S6).

The orientation results for the PFOA–semicrystalline PE system reveal that upon crossing the shaded interfacial region (gray area) and moving toward the PE matrix, a sharp increase in orientational order is observed. This behavior indicates a transition from a more disordered configuration, likely influenced by interactions with interfacial water molecules, to a more ordered state within the PE slab. In this region, the orientational order parameter reaches approximately 0.78 at ξ ≈ −4.7 Å, reflecting a preferential alignment of the PFOA backbone relative to the polymer chains (Figure 4A). This behavior is further supported by Figure S6A, which shows the distribution of orientation angles centered at around 10°. Beyond the interface (ξ > 0), *S*_*v*_ fluctuated around zero, consistent with isotropic orientations in bulk water. The PMF result (Figure 3A) demonstrated that adsorption onto the surface does not prevent the molecule from exploring a wide range of orientations. Snapshots obtained during the PMF simulations at different separation distances between PFOA and the PE–water interface are also exhibited in Figure 5A.

**Figure 5:**
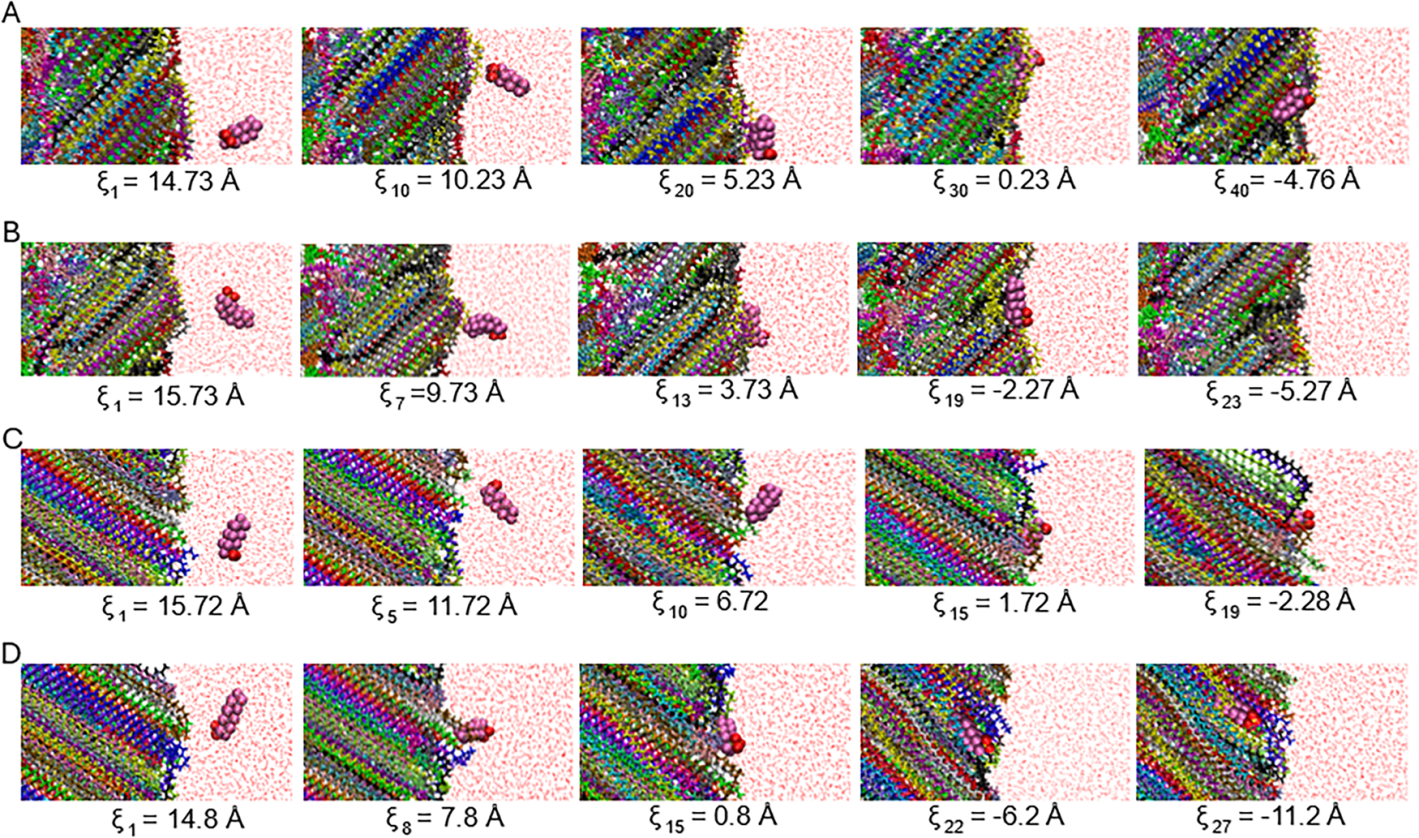
Snapshots obtained during the PMF simulations at different separation distances between PFAS and the PE–water interface: (A) PFOA in the semicrystalline PE box, (B) PFOS in the semicrystalline PE box, (C) PFOA in the crystalline PE box, and (D) PFOS in the crystalline PE box.

For the PFOA–crystalline PE system (Figures 4C and 5C), prior to the Gibbs dividing surface, at distances around ξ ≈ 2 Å toward the PE slab, a pronounced increase in orientational ordering can be observed with *S*_*v*_ values reaching approximately 0.77 at ξ = −2.3 Å. As shown in Figure S6C, PFOA was not perfectly aligned over time, although most had already adopted a similar orientation. This enhanced alignment coincides with the region where the PMF curve departs from its minimum and begins to rise toward higher free-energy values, indicating that the increasing orientational constraint contributes to the free-energy penalty associated with further penetration into the PE phase. This behavior likely reflects the stronger structural constraints imposed by the more ordered crystalline packing, which not only hinder PFOA insertion into the slab but also restrict the diversity of accessible molecular orientations, as corroborated by the PMF profile (Figure 3B).

Regarding intramolecular conformational changes, PFOA exhibits only small RMSD fluctuations across all investigated systems, including both crystalline and semicrystalline PE. This behavior indicates that PFOA largely retains an extended backbone conformation throughout the simulations, with no evidence of significant intramolecular rearrangements (Figure S7A,C).

In the PFOS–semicrystalline PE system (Figures 4B and 5B), PFOS initially exhibits a tendency to align parallel to the polymer chains near the Gibbs dividing surface. As it penetrates further into the PE slab, the molecule progressively shifts toward a more perpendicular orientation. This change in orientational preference is mirrored in the PMF profile, where the small barriers inside the slab are associated with transitions in the molecular orientation (Figures 3A and S6B).

Analysis of the PFOS–crystalline PE system suggested that PFOS tends to enter the interfacial region (shaded area) from water toward the PE slab in an almost perpendicular orientation, likely driven by the hydrophobic nature of its fluoroalkyl tail combined with the polarity and size of the sulfonate functional group. The Gibbs dividing surface showed a tendency for parallel alignment, and within the slab, *S*_*v*_ remained close to 1, indicating strong alignment of PFOS tails with the PE chains (Figures 4D and 5D). Notably, as PFOS aligned parallel to the PE chains, an increase in PMF was observed (Figures 3B and S6D).

In contrast to PFOA, PFOS exhibits a broader RMSD distribution, with deviations reaching up to approximately 1.5 Å, indicating access to nonfully extended configurations. In the semicrystalline PE system, PFOS samples multiple conformations even within the polymer slab (Figure S7B), whereas in crystalline PE the conformational variability becomes more restricted at positions deeper inside the slab (ξ ≤ −10 Å), consistent with the stronger structural constraints imposed by the ordered polymer matrix. Moreover, near the Gibbs dividing surface, PFOS preferentially adopts more extended conformations compared with those sampled in bulk water (Figure S7D).

Overall, these results demonstrate that PFAS molecules preferentially align their tails with PE chains when embedded within the polymer slab, while orientation is disrupted upon the transition to bulk water. Crystalline PE generally promotes more stable alignment compared to semicrystalline PE, reflecting the influence of chain packing and the free volume. PFOS in the semicrystalline PE system represents a notable exception: instead of maintaining strong tail alignment within the slab, it exhibits a more perpendicular orientation. This behavior is consistent with the small barriers observed in the PMF profile, indicating that molecular reorientation within the amorphous domains contributes to the weaker stabilization of the PFOS alignment in this morphology.

Understanding these interactions can support the development of strategies for PFAS removal and containment. For instance, polyethylene has already been employed in passive samplers designed to detect neutral polyfluorinated alkyl substances in air and water.^69^ Recent work has also explored the use of HDPE geomembranes to minimize PFAS migration, showing that intact HDPE barriers can significantly slow the transport of these contaminants.^67^

In such applied contexts, the study of PFAS aggregation and micellization may also become relevant. However, experimental evidence reported by Klevan et al.^70^ indicates that, at least for PFOA, micelle formation in solution is unlikely under environmentally relevant concentrations. Accordingly, these collective effects are beyond the scope of the present work, which focuses on highly dilute conditions. Importantly, surface-induced micellization or hemimicelle formation is not expected on polyethylene surfaces, as PE is electrically neutral and hydrophobic. Such aggregation phenomena have been primarily associated with positively charged adsorbent surfaces.^71^

## Conclusions

In this study, we employed molecular dynamics simulations to investigate the interactions of two representative PFAS, perfluorooctanoic acid (PFOA) and perfluorooctanesulfonic acid (PFOS), with polyethylene (PE) slabs, providing molecular-level insights into how these organic contaminants interact with microplastics.

Randomly coiled PE chains evolved into a semicrystalline morphology with lamellar and amorphous regions, whereas extended chains formed a more crystalline structure, both representative of HDPE. Density profiles confirmed this distinction, with semicrystalline PE showing a lower density (0.93 g cm^–3^) than crystalline PE (0.99 g cm^–3^), and enabled the identification of the Gibbs dividing surface for analyzing the PE–water interface.

Potential of mean force calculations indicated that, within statistical uncertainty, the depth of the free-energy minimum is comparable for semicrystalline and crystalline PE. PFOS nonetheless interacts more strongly with PE than PFOA. We interpret this enhanced affinity as arising from a combined effect of the longer perfluoroalkyl chain and the bulkier sulfonate headgroup of PFOS. However, an unequivocal separation of chain length and headgroup contributions would require additional studies employing deprotonated PFAS with systematically varied molecular structures, which would enable a more detailed understanding of the mechanisms governing PFAS–PE interactions.

Furthermore, orientation analyses revealed preferential tail alignment of PFAS with PE chains inside the slab, which was disrupted upon the transition to bulk water. PFOS in the semicrystalline system represented a distinct case, exhibiting a tendency toward perpendicular orientations within the slab, in agreement with the small barriers observed in the PMF profile.

Importantly, under the dilute conditions investigated and given the electrically neutral and hydrophobic nature of polyethylene, surface-induced micellization or hemimicelle formation is not expected. Consequently, the observed free-energy profiles and orientational behaviors can be attributed to single-molecule PFAS–PE interactions rather than aggregation phenomena.

Overall, the results indicate that PFAS characteristics influence PFAS–PE interactions, with microplastics serving as reservoirs that can enhance the persistence and transport of PFAS in aquatic environments. While this poses environmental risks, the same molecular insights may be leveraged to design PE-based materials for PFAS monitoring and remediation.

## Supplementary Material



## Notes

Force field tables, density profiles, umbrella sampling details, angle of interaction between PFAS and PE, and root-mean-square deviation of atomic positions (PDF)
