# Supplementary material for: Molecular-Scale Insights into the Interactions between Perfluoroalkyl Substances and Polyethylene
Source: J Phys Chem B. 2026 Mar 5;130(11):3206–16. doi: 10.1021/acs.jpcb.5c06774 (PMC13007032; doi:10.1021/acs.jpcb.5c06774)
Supplement: Supplementary file 1 [file jp5c06774_si_001.pdf]

Supporting Information
Molecular-Scale Insights into the Interactions between Perfluoroalkyl Substances and Polyethylene

Dandara Freitas Thomaz, Eduardo Rocha de Almeida Lima, and Nathalia Salles Vernin*

Chemical Engineering Graduate Program, Rio de Janeiro State University, Rio de Janeiro, RJ 20550-900, Brazil

E-mail: nathalia.vernin@uerj.br

1 Force Field parameter for PFOA and PFOS

The OPLS-AA parameters are expressed in terms of the atom type. Fluorine is of type f1, the carbon bond to fluorine is of type c1, the oxygen of the carboxylate is of type oc, the carbon of the carboxylate is of type c2, the sulfur from sulfonate is of type s1, and the oxygen from the sulfonate is of type os.

Table S1: OPLS-AA Lennard-Jones parameters for PFAS.

Atom type	ϵ_{ii} (kcal mol^{-1})	σ_{ii} (Å)
c1	0.066	3.500
f1	0.060	2.900
oc	0.210	2.960
os	0.170	2.960
c2	0.070	3.550
s1	0.250	3.550

Table S2: OPLS-AA harmonic bond parameters for PFAS.

Bond type	$k_{b,i}$ (kcal mol^{-1} Å^{-2})	r_{i0} (Å)
f1 – c1	367.000	1.360
c1 – c1	268.000	1.529
c2 – oc	656.000	1.250
c2 – c1	317.000	1.522
c1 – s1	340.000	1.770
s1 – os	700.000	1.440

Table S3: OPLS-AA harmonic angle parameters for PFAS.

Angle type	K (kcal mol^{-1} degree^{-2})	θ_0 (degree)
f1 – c1 – f1	77.000	109.100
f1 – c1 – c1	50.000	109.500
c1 – c1 – c1	58.350	112.700
c1 – c2 – oc	70.000	117.000
oc – c2 – oc	80.000	126.000
c1 – c1 – c2	63.000	111.100
c2 – c1 – f1	35.000	109.500
c1 – c1 – s1	50.000	114.700
c1 – s1 – os	74.000	108.900
os – s1 – os	104.000	119.000
os – c1 – f1	50.000	109.500

Table S4: **OPLS-AA parameters for the dihedrals of PFAS.**

Dihedral type	$V_{1,i}$ (kcal mol^{-1})	$V_{2,i}$ (kcal mol^{-1})	$V_{3,i}$ (kcal mol^{-1})
c1 – c1 – c1 – c1	0.000	7.250	0.000
f1 – c1 – c1 – f1	-2.500	0.000	0.250
c2 – c1 – c1 – c1	-2.060	-0.313	0.0315
c1 – c1 – c1 – s1	1.262	-0.198	0.465
f1 – c1 – c1 – s1	0.000	0.450	0.000

Table S5: **OPLS-AA parameters for the improper of PFOA.**

Improper type	k (kcal mol^{-1})	d	n
c2 – c1 – o1 – o1	10.500	-1	2

Regarding the partial atomic charges (Tables S6 e S7), each atom possesses a value. Therefore, Figure S1 shows the identification of each atom of PFOA, whereas Figure S2 refers to PFOS.

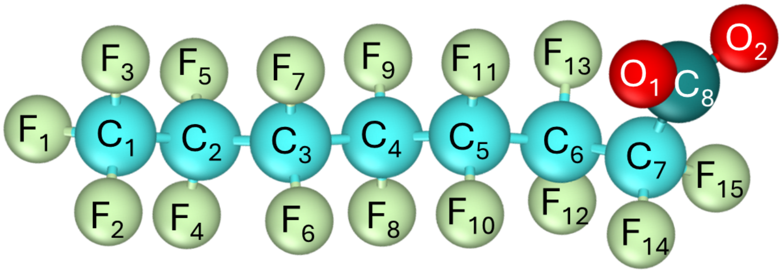

Figure S1: PFOA structure and identification of the atoms.

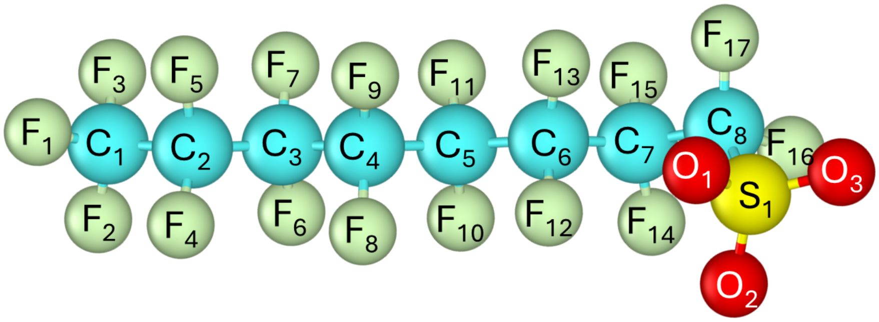

Figure S2: PFOS structure and identification of the atoms.

Table S6: Partial atomic charges for PFOA.

Atom	q_i	Atom	q_i
C1	0.3381	C5	0.1825
F1	-0.1188	F10	-0.0759
F2	-0.1188	F11	-0.0759
F3	-0.1188	C6	0.2022
C2	0.1387	F12	-0.1351
F4	-0.0833	F13	-0.1351
F5	-0.0833	C7	0.1804
C3	0.1499	F14	-0.1393
F6	-0.0821	F15	-0.1393
F7	-0.0821	C8	0.3935
C4	0.1405	O1	-0.5769
F8	-0.0921	O2	-0.5769
F9	-0.0921		

Table S7: Partial atomic charges for PFOS.

Atom	q_i		
C1	0.3375	F11	-0.0986
F1	-0.1162	C6	0.1426
F2	-0.1162	F12	-0.1050
F3	-0.1162	F13	-0.1050
C2	0.1471	C7	0.2339
F4	-0.0741	F14	-0.0953
F5	-0.0741	F15	-0.0953
C3	0.1519	C8	-0.2099
F6	-0.0827	F16	-0.0989
F7	-0.0827	F17	-0.0989
C4	0.1604	S1	12.951
F8	-0.0789	O1	-0.5995
F9	-0.0789	O2	-0.5995
C5	0.1555	O3	-0.5995
F10	-0.0986		

2 Simulation Boxes

Representative configurations of PFAS molecules positioned 15 Å from the polyethylene–water interface are presented in Figure S3.

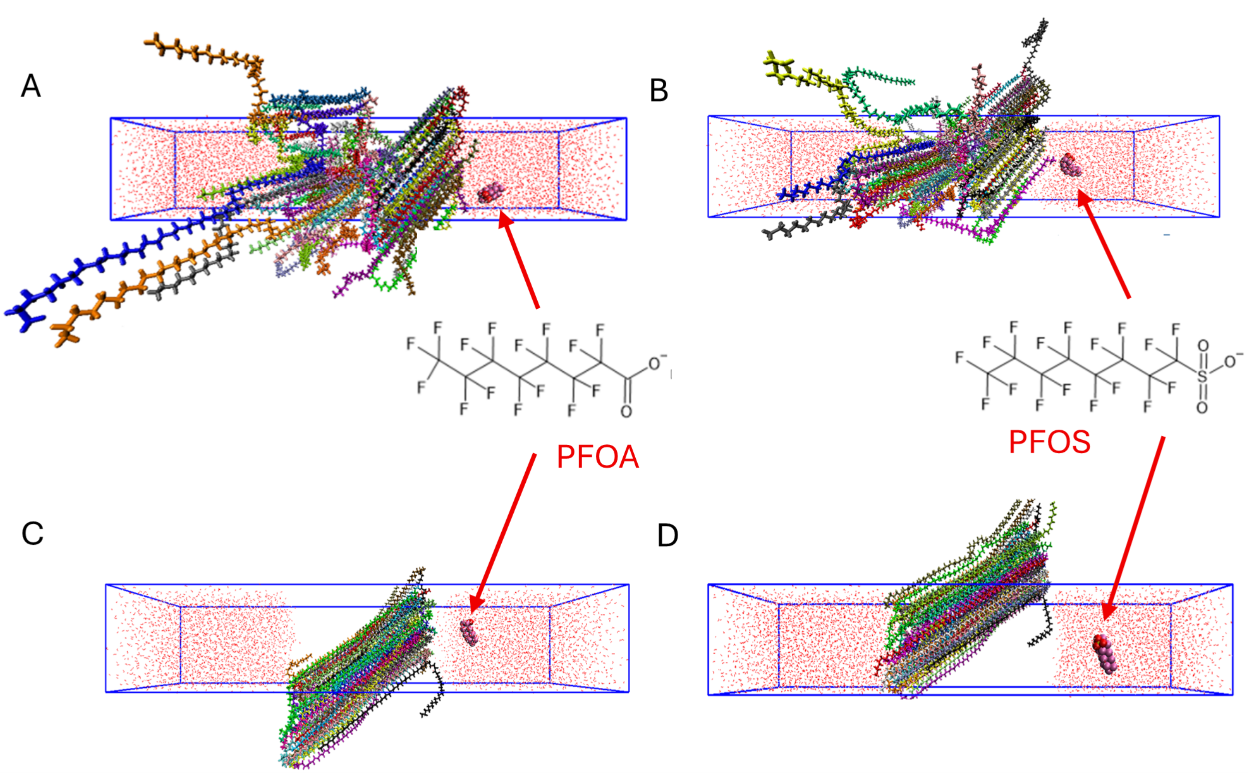

Figure S3: PFAS molecules positioned 15 Å from the polyethylene–water interface: (A) semicrystalline PE with PFOA; (B) semicrystalline PE with PFOS; (C) crystalline PE with PFOA; (D) crystalline PE with PFOS. The molecular coordinates were wrapped within the central simulation cell. However, the intramolecular connectivity was preserved, such that atoms belonging to the same molecule were not arbitrarily split across periodic boundaries. Consequently, the apparent ”voids” observed within the PE slab are not genuine empty regions. Rather, they correspond to PE atoms whose molecules extend across the boundaries of the central box and are thus represented in adjacent periodic replicas.

3 Density Profiles

The density profiles of water and polyethylene for the four systems are reported in Figure S4 and were used to locate the Gibbs dividing surface at 34.7 Å (PFOA–semicrystalline PE system), 34.7 Å (PFOS–semicrystalline PE system), 35.3 Å (PFOA–crystalline PE system), and 35.2 Å (PFOS–crystalline PE system).

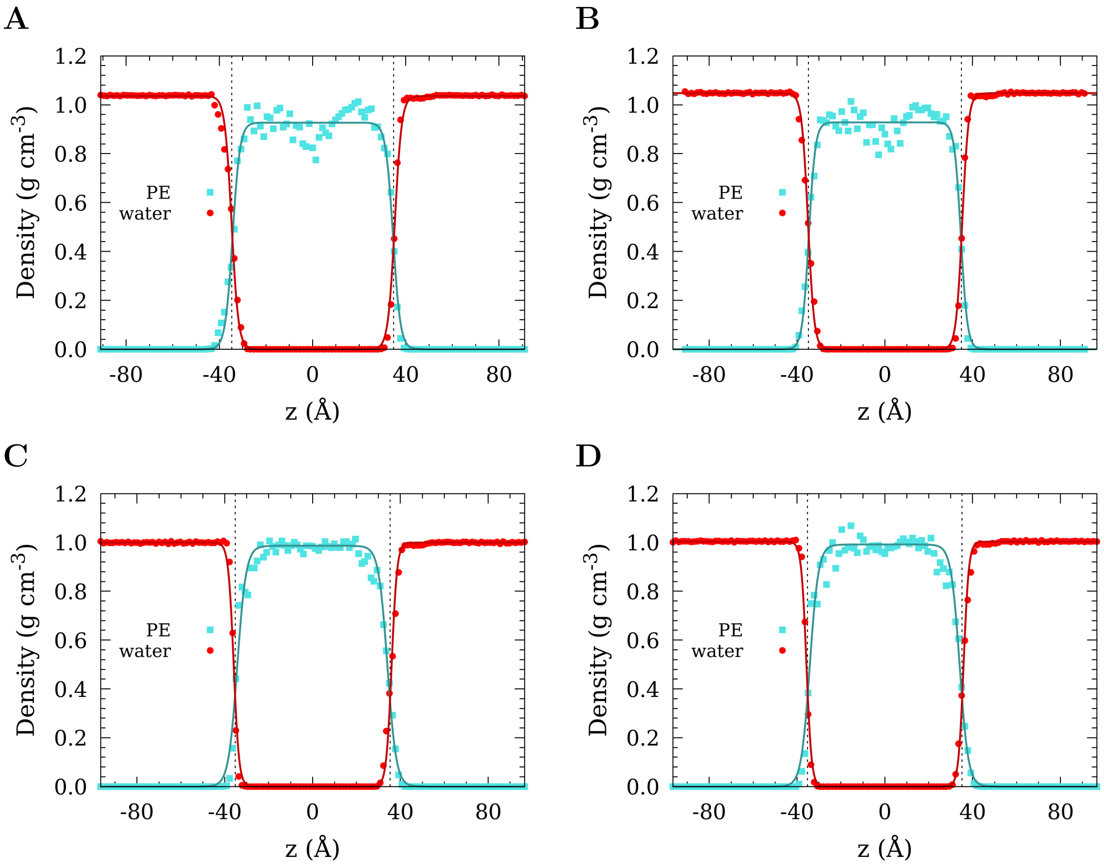

Figure S4: Density profiles of water and PE for the systems (A) PFOA in semicrystalline PE, (B) PFOS in semicrystalline PE, (C) PFOA in crystalline PE, (D) PFOS in crystalline PE.

4 Histograms from Umbrella Sampling

Representative histograms obtained from the umbrella sampling simulations for PFOA and PFOS are shown in Figure S5. Additional replicas were used for statistical analysis but are not shown. These distributions were used as input for the WHAM analysis to generate the potential of mean force (PMF) profiles presented in the main text.

For the PFOA–semicrystalline PE system (Figure S5A), 40 windows were used with centers spaced 0.5 Å apart, applying a force constant of 10 kcal mol^{-1} Å^{-2} in all cases, starting at ξ equal to 15.2 Å toward the PE slab.

For the PFOS–semicrystalline PE system, 23 windows were employed with centers spaced 1.0 Å apart, starting at ξ equal to 15.7 Å toward the PE slab. A force constant of 10 kcal mol^{-1} Å^{-2} was applied to all windows, except for that indicated explicitly in Figure S5B, where both the center of the window ξ_i and the force constant were adjusted.

For the PFOA–crystalline PE system (Figure S5C), 19 windows were used with centers spaced 1.0 Å apart, applying a force constant of 10 kcal mol^{-1} Å^{-2} in all cases, starting at ξ equal to 15.7 Å toward the PE slab.

For the PFOS–crystalline PE system, 27 windows were employed with centers spaced 1.0 Å apart, starting at ξ equal to 15.8 Å toward the PE slab. A force constant of 10 kcal mol^{-1} Å^{-2} was applied to all windows, except for one window specifically indicated where the force constant was adjusted (Figure S5D).

5 Angle of interaction between PFAS and PE

The boxplots of the interaction angle between PFAS and PE as a function of the reaction coordinate are shown in Figure S6.

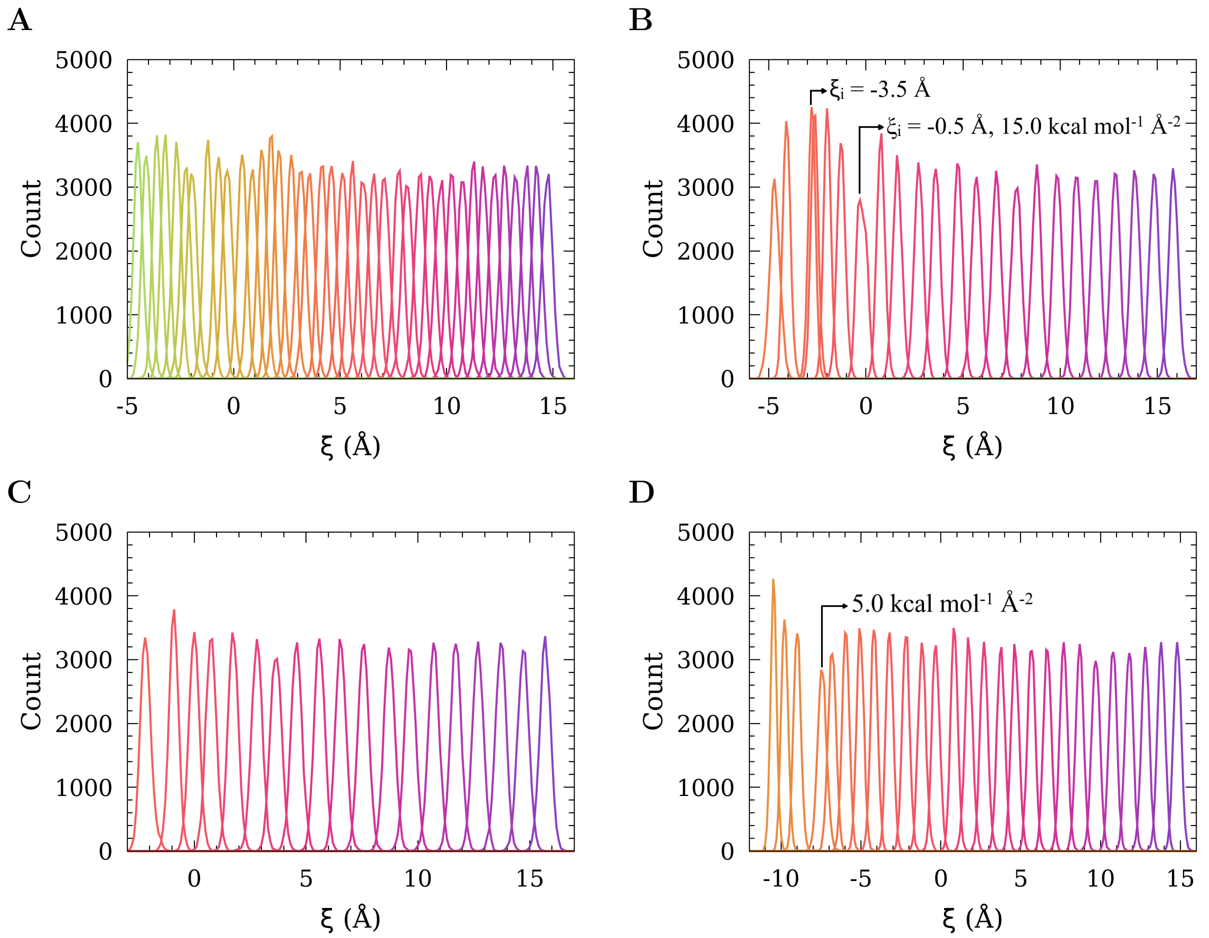

Figure S5: Representative histograms collected from umbrella sampling windows for the systems (A) PFOA in semicrystalline PE, (B) PFOS in semicrystalline PE, (C) PFOA in crystalline PE, and (D) PFOS in crystalline PE as a function of the reaction coordinate.

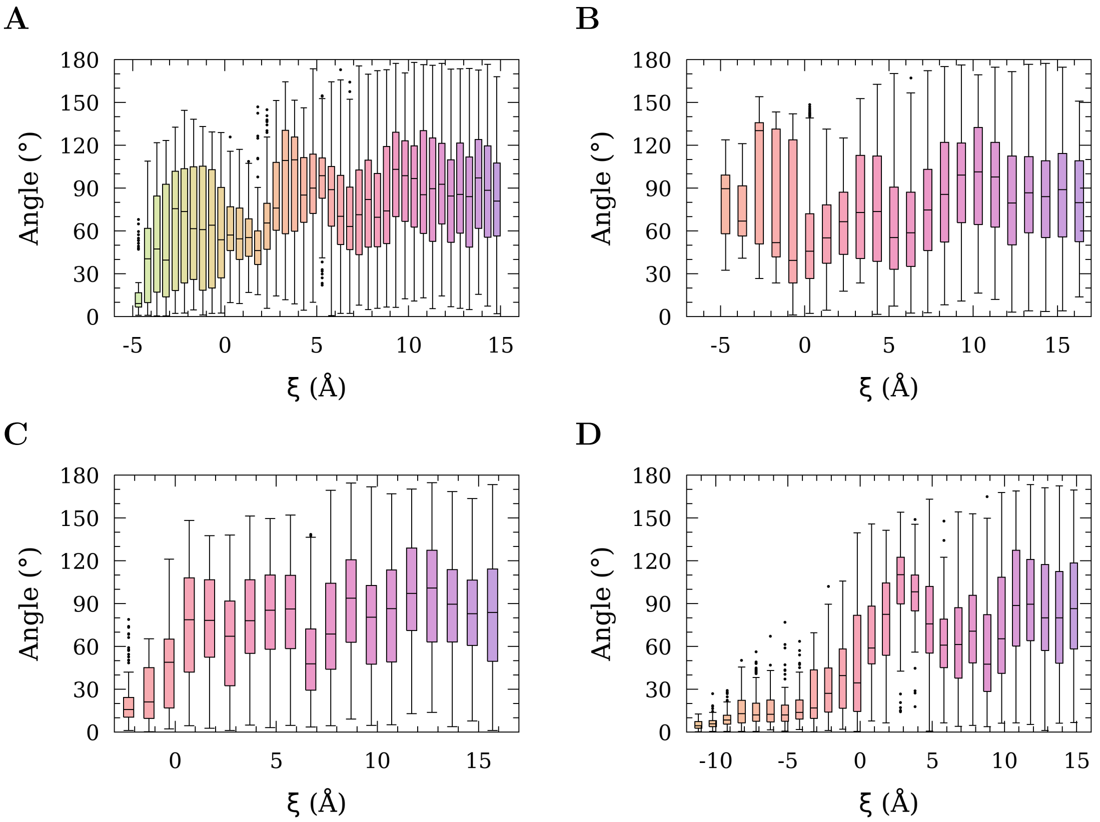

Figure S6: Boxplots of the interaction angle between (A) PFOA and semicrystalline PE, (B) PFOS and semicrystalline PE, (C) PFOA and crystalline PE, and (D) PFOS and crystalline PE as a function of the reaction coordinate.

6 Root mean square deviation of atomic positions

Figure S7 presents the boxplots for root-mean-square deviation (RMSD) of the atomic positions of carbon and sulfur atoms in the PFAS molecules as a function of the reaction coordinate.

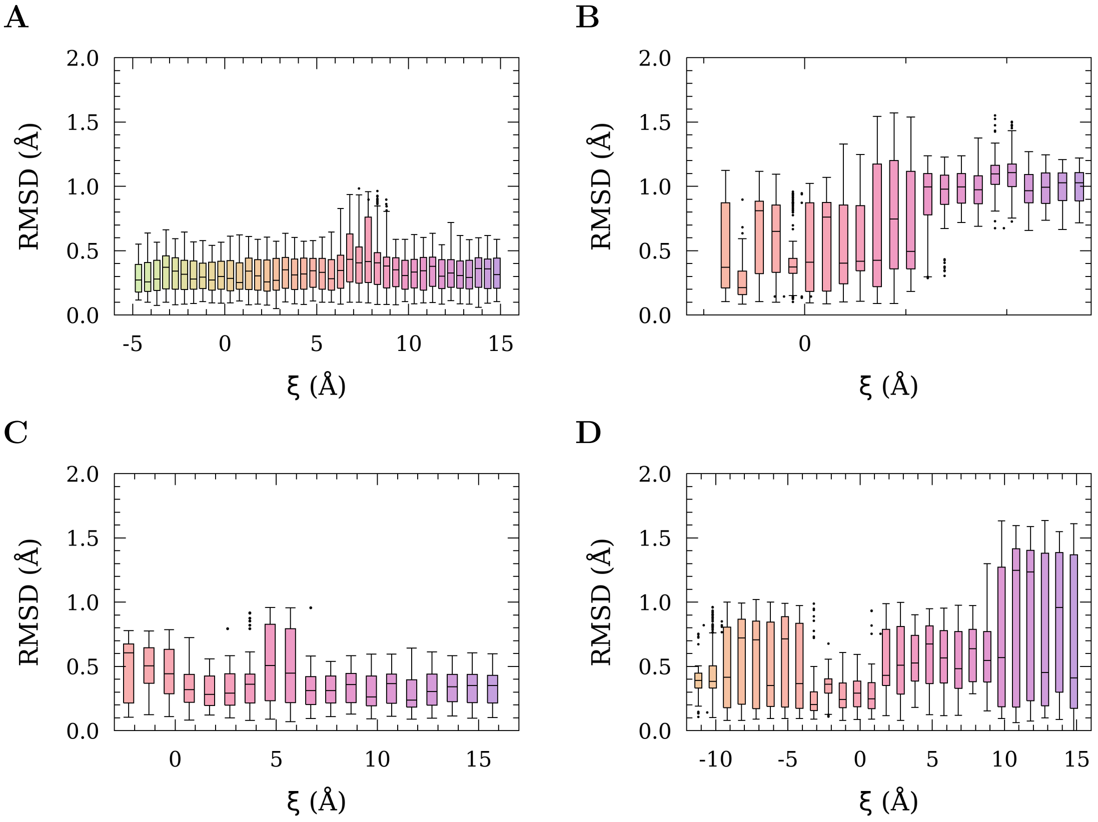

Figure S7: Boxplot of RMSD of the atomic positions of carbon and sulfur atoms in the PFAS molecules for the systems (A) PFOA in semicrystalline PE, (B) PFOS in semicrystalline PE, (C) PFOA in crystalline PE, (D) PFOS in crystalline PE as a function of the reaction coordinate.
